# Vestibular Schwannoma Surgery with Endoscope-Assisted Retrolabyrinthine Approach under Modified Reinforced Continuous Intraoperative Monitoring for Hearing Preservation: Experience of 33 Cases in a Single Center

**DOI:** 10.3390/diagnostics13020275

**Published:** 2023-01-11

**Authors:** Makoto Hosoya, Takanori Nishiyama, Takeshi Wakabayashi, Marie N Shimanuki, Hidemi Miyazaki, Hiroyuki Ozawa, Naoki Oishi

**Affiliations:** Department of Otolaryngology, School of Medicine, Keio University, 35 Shinanomachi, Shinjuku-ku, Tokyo 160-8582, Japan

**Keywords:** vestibular schwannoma, hearing preservation surgery, retrolabyrinthine approach, DNAP, monitoring

## Abstract

Surgery for vestibular schwannoma includes various techniques such as the middle cranial fossa, suboccipital, translabyrinthine, and retrolabyrinthine approaches. The retrolabyrinthine approach does not impair the labyrinth and allows postoperative hearing preservation. Previously, we reported an endoscope-assisted retrolabyrinthine approach under reinforced continuous intraoperative monitoring for preservation of hearing and facial nerve function. However, the hearing preservation rate was relatively low in patients with long-wave V latency in the auditory brainstem response or poor otoacoustic emission response under this approach. Thus, the hearing preservation rate in such patients remains to be improved. To overcome this limitation, we modified the previous approach. In 26 of the 33 consecutive cases (79%) over the last three years, preservation of hearing equivalent to that before surgery or improved hearing was achieved. Postoperative deafness was observed in only two of the 33 cases (6%). According to previous reports, the rate of hearing preservation using the retrolabyrinthine approach is approximately 30–50%. Therefore, we have achieved a higher hearing preservation rate than that previously reported using the endoscopy-assisted retrolabyrinthine approach under reinforced continuous intraoperative monitoring. These improvements in our department are considered to be helpful for hearing preservation.

## 1. Introduction

Vestibular schwannoma is a benign tumor originating from the Schwann cells of the auditory nerve, especially the inferior vestibular nerve [1]. Tumors can impair the auditory nerve and lead to hearing loss. Moreover, vestibular schwannomas can affect the facial nerve, which runs parallel to the auditory nerve in the internal auditory canal, resulting in facial nerve palsy. Therefore, vestibular schwannomas can harm quality of life by affecting both hearing ability and facial nerve function [2,3].

Although tumor growth can impair a patient’s hearing, it is possible to preserve hearing with several treatment modalities, including surgical resection and radiation therapy, before significant damage is caused to the auditory or facial nerves. Unfortunately, the treatment of vestibular schwannoma itself can cause hearing loss. Therefore, because of the potential side effects of treatment and the fact that the tumor is benign and non-invasive, a “wait and scan” policy may be chosen, especially for patients without hearing loss or facial nerve palsy.

Surgery for vestibular schwannoma includes various techniques such as the middle cranial fossa, suboccipital, translabyrinthine, and retrolabyrinthine approaches. Surgical interventions for vestibular schwannomas can be categorized into two types: non-hearing preservation surgery and hearing preservation surgery. Most non-hearing preservation surgeries are performed using the translabyrinthine approach [4]. The transpromontorial approach can also be used in several cases [5]. Other surgical methods can be used in cases of hearing preservation, including the retrosigmoid, middle cranial fossa, and retrolabyrinthine approaches. The selection of the surgical approach depends on the degree of residual hearing, tumor size, tumor location, and whether hearing preservation is preferred.

If hearing preservation surgery is selected, preservation of hearing should be the central focus of the procedure. Moreover, irrespective of the surgical category, surgeons must attempt to prevent facial nerve palsy, particularly in cases with no evident preoperative deficit. Therefore, intraoperative neural monitoring is widely used to assess postoperative hearing deficits and facial nerve function.

In our department, we have established an endoscope-assisted retrolabyrinthine approach under reinforced continuous intraoperative monitoring for hearing preservation and facial nerve function [6,7]. The retrolabyrinthine surgical approach does not impair the labyrinth and allows postoperative hearing preservation [8,9,10]. This technique is less invasive than the middle cranial fossa and the suboccipital approaches. Unfortunately, the surgical field is relatively narrower than that of other approaches, and the fundus of the inner ear canal cannot be visualized by surgical microscopy alone. However, an endoscope can overcome this limitation to a certain extent. In the endoscope-assisted retrolabyrinthine approach under reinforced continuous intraoperative monitoring, electrodes can be placed near the dorsal cochlear nerve nucleus for more advanced monitoring of cochlear nerve function (dorsal cochlear nucleus activation potential [DNAP] monitoring) and the root exit zone of the facial nerve to monitor facial nerve function (facial nerve root evoked muscle activation potential [FREMAP] monitoring) [6,11,12]. 

Previously, the hearing preservation rate was relatively low in patients with long auditory brainstem (ABR) wave V latency or poor otoacoustic emission (OAE) response using the endoscope-assisted retrolabyrinthine approach under reinforced continuous intraoperative monitoring [6]. In contrast, a high hearing preservation rate has been achieved in patients without elongation of ABR wave V latency or accompanying good OAE responses. Therefore, improving the hearing preservation rate in patients with elongation of ABR wave V latency and poor OAE responses remains a challenge. 

**Figure 1:**
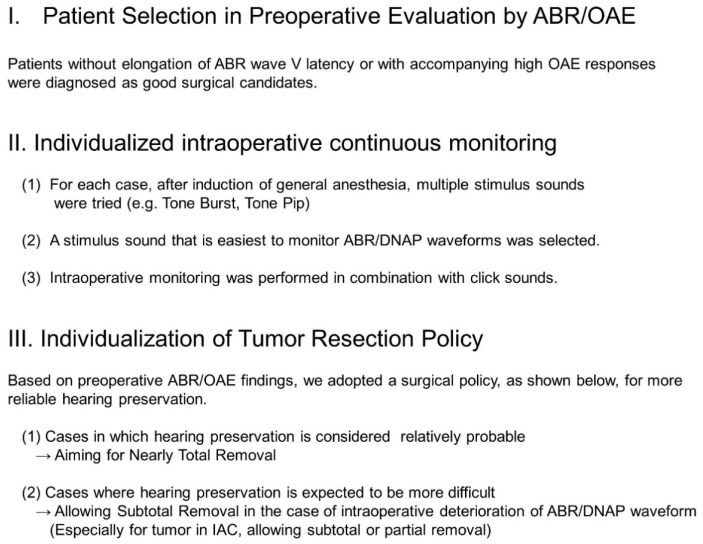
Surgical concepts of endoscope-assisted retrolabyrinthine approach under modified reinforced continuous intraoperative monitoring for hearing preservation in our hospital. We have added modifications and improvements to our previous methods of patient selection, monitoring methods, and tumor resection policies. ABR, auditory brainstem; DNAP, dorsal cochlear nucleus activation potential; OAE, otoacoustic emission; IAC, internal auditory canal.

To overcome this limitation, we modified the previous approach (Figure 1). In this modified approach, an “individualized intraoperative continuous monitoring” method for retrolabyrinthine approach cases is applied, in which routine click monitoring is difficult or postoperative hearing preservation is expected to be more difficult. Moreover, we set different resection policies for these patients to maximize the possibility of preservation of hearing. In this study, we report recent experiences in our hospital.

## 2. Materials and Methods

### 2.1. Enrollment

Cases were recruited from Keio University Hospital, and a single surgeon performed all surgical procedures. 

Based on our previous study, patients without elongation of ABR wave V latency or with accompanying high OAE responses were diagnosed as good surgical candidates [6]. In contrast, patients with elongation of the ABR wave V latency and poor OAE responses were diagnosed as not ideal surgical candidates. In this case, whether surgery should be performed depends on the patient’s requirement for hearing preservation surgery and the acceptance of the possibility of partial resection or hearing loss caused by surgical interventions.

Patients with vestibular schwannoma who underwent retrolabyrinthine surgery between April 2019 and June 2022 were enrolled (N = 33). All 33 patients underwent reinforced continuous intraoperative monitoring with FREMAP and DNAP to monitor facial nerve function and preserve hearing.

The following parameters were examined:

(1) Pre- and postoperative hearing function (including pure tone audiometry and speech audiometry) and preoperative OAE and ABR. Pre- and postoperative hearing function was assessed using a standardized scattergram format. Postoperative hearing function was assessed at three months after surgery. For the present analyses, hearing preservation was defined as a postoperative decrease of ≤20 dB on pure tone audiometry and ≤20% on speech audiometry, as previously reported [6].
(2) The degree of tumor resection was defined as follows: (i) total resection: tumor completely resected except the capsule; (ii) near total resection: >95% resection; (iii) subtotal resection: >90% resection; and (iv) partial resection: <90% resection [13,14,15].

### 2.2. Intraoperative Monitoring

**Figure 2:**
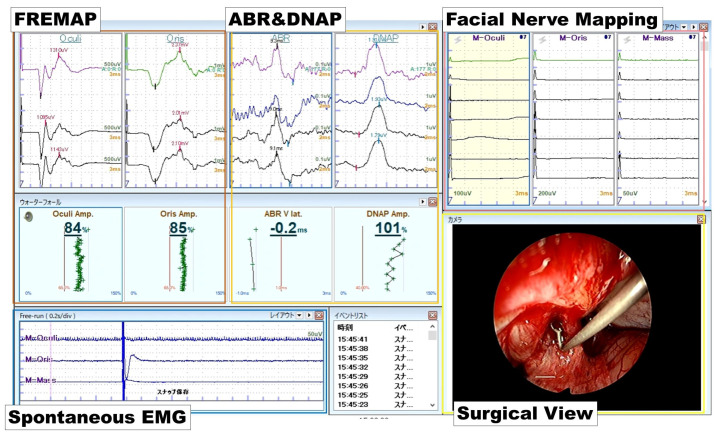
View of the intraoperative monitoring system. In this screen, the monitoring team can get information on surgical view, spontaneous EMG, FREMAP, ABR, and facial nerve mapping at a glance. In case of deterioration of the waveform is observed, the monitoring team would caution the surgeon. EMG: electromyography.

The surgeon attempted to maintain the ABR waveform and DNAP amplitude at >40% of the initial measurement throughout the surgery. If the amplitude decreased during the tumor resection, the surgeon stopped the procedure or selected an alternative resection route. After recovery of the amplitude, the tumor resection was continued. An example of intraoperative monitoring view is shown in Figure 2. 

In the case of patients with unclear intraoperative ABR wave V waveform by standard click sound stimulation, we tried to create and use another sound stimulation by which clearer wave V waveforms could be observed in each patient. Stimulation sounds were optimized by modifying the rise/fall time, plateau time, and frequency of the sound based on tone pips or tone burst sounds. Once the sound source that could induce a clearer wave V was obtained, the sound was used for continuous intraoperative monitoring (“individualized intraoperative continuous monitoring”). This modification of the stimulation sound was performed after general anesthesia and before starting tumor resection. 

### 2.3. Surgical Procedure

The surgery was performed as previously reported [6]. A U-shaped skin incision was made over the temporal mastoid. Following mastoidectomy and skeletonization of the sigmoid sinus, the bony wall of the sigmoid sinus was partially removed. The exposed sigmoid sinus was then pushed down, and the temporal bone was resected between the posterior semicircular canal and posterior fossa. After drilling the bone around the internal auditory canal, the dura was opened and the tumor was identified. The FREMAP and DNAP electrodes were placed on the facial nerve root and dorsal cochlear nucleus.

The value of the endoscope has been reported in previous studies of vestibular schwannoma surgery in which it was applied in combination with a retrolabyrinthine approach [16,17,18]. The use of an angled endoscope (30°) was combined with microscopic resection if indicated. At the end of the procedure, the FREMAP and DNAP electrodes were removed, the dura was closed using the temporalis muscle fascia and fibrin glue, and the temporal bone cavity was filled with abdominal fat.

## 3. Results

**Figure 3:**
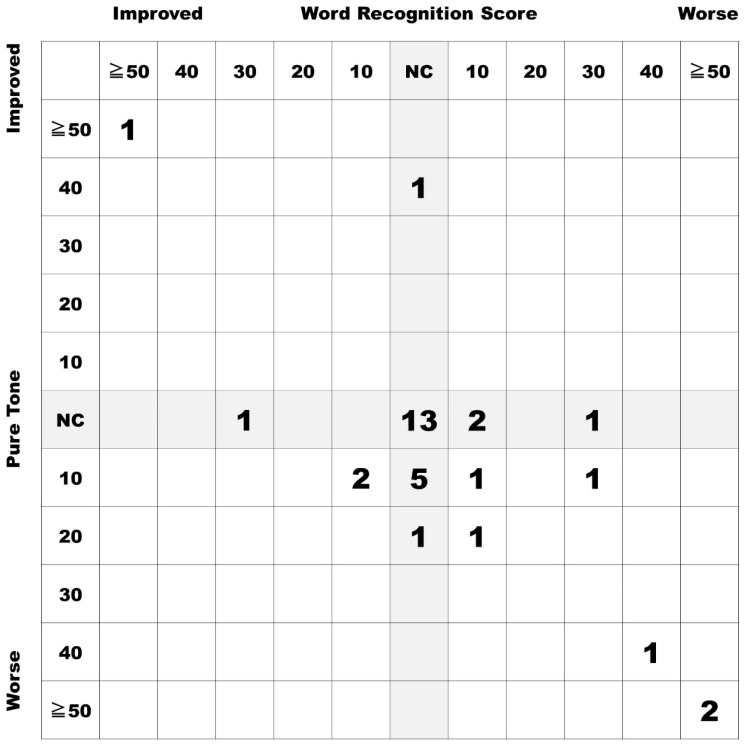
Postoperative hearing results of included cases in this study. In 26 of the 33 cases (79%), preservation of hearing was equivalent to that before surgery or improved hearing. Only two cases of deafness (6%) occurred after surgery. NC, No change.

Thirty-three patients (13 males, 20 females) were included in this study. The average age of the patients at the surgery was 47.3 ± 13.7 years old. The average size of the tumors was 17.4 ± 5.7 mm (Koos Classification; I (N = 4), IIa (N = 18), IIb (N = 2), III (N = 8), and IV (N = 1)). In 26 of the 33 cases (79%), preservation of hearing equivalent to that before surgery or improved hearing was achieved (Figure 3). In five of the seven residual cases, some extent of hearing remained, and total deafness was present in only two of the 33 cases (6%).

**Figure 4:**
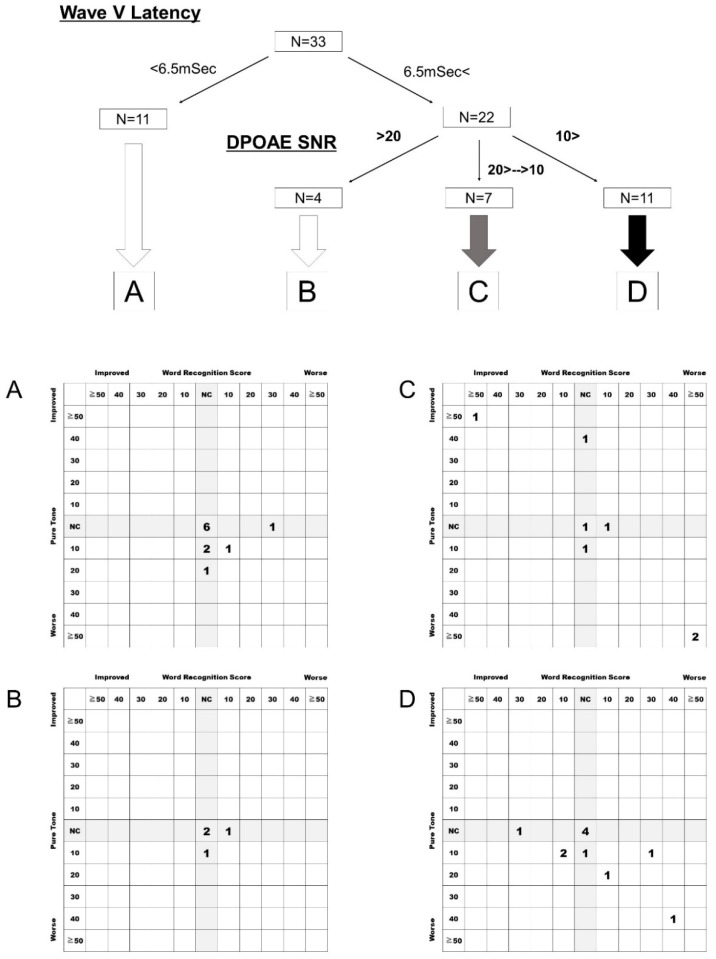
Postoperative hearing preservation rate based on our previous classification. In class A patients, hearing preservation was achieved in nine of 11 patients (82%). In class B, four of four patients (100%) had preserved preoperative hearing levels. In class C, five of seven (71%) patients achieved hearing preservation, while two of seven lost hearing ability. In class D, eight of 11 patients (73%) had preserved hearing levels postoperatively. NC, No change.

Next, the postoperative hearing results were analyzed based on the classification suggested in our previous report of ABR wave V latency and OAE (Figure 4). In class A patients, hearing preservation was achieved in nine of 11 patients (82%). In class B, four of four patients (100%) had preserved preoperative hearing levels. In class C, five of seven (71%) patients achieved hearing preservation, while two of seven lost hearing ability. In class D, eight of 11 patients (73%) had preserved hearing levels postoperatively.

Finally, we analyzed the extent of tumor resection in each patient. Total tumor resection or near-total resection was achieved in 18 of 33 patients (55%) (Table 1). Based on our previous classification, total tumor resection was performed only in class A patients. In class A or B patients, total tumor resection or near total resection was achieved in 10 of 15 patients (67%), while near-total resection was achieved in eight of 18 patients (44%) in class C or D patients. No postoperative facial nerve disturbance during tumor resection was observed in any patient. No other postoperative serious complications, including permanent complications with the central nervous system, nor mortality was observed in any patient.

## 4. Discussion

To improve the postoperative hearing preservation rate in vestibular schwannoma surgery, we attempted to modify the retrolabyrinthine approach under reinforced continuous intraoperative monitoring for vestibular schwannoma by modifying our previous approach [6,7]. The modification included improvement in preoperative evaluation, personalization of intraoperative monitoring, and resection policy depending on preoperative evaluations. Compared to our previous report [6], in which only 25% of class C patients and 0% of class D patients achieved hearing preservation, this study showed that hearing preservation was achieved in approximately 71% (class C) and 73% (class D) of patients. Therefore, these modifications resulted in high hearing and relatively high preservation rates compared to those in our previous study.

According to previous reports, the rate of hearing preservation using the retrolabyrinthine approach is approximately 22–50% [10,19,20]. Bento et al. recently reported a hearing preservation rate of 49.7% [10]. In the present study, we observed an overall hearing preservation rate of 79%. Moreover, when limited to more selected patients, >80% hearing preservation was achieved (patients in class A or B). Therefore, a higher hearing preservation rate was achieved compared to previous studies, accompanied by an endoscopy-assisted retrolabyrinthine approach under reinforced continuous intraoperative monitoring with these modifications, which adds patient selection following preoperative evaluation using ABR/OAE, individualizing a tumor removal strategy, and continuous monitoring using ABR/DNAP to the retrolabyrinthine approach. These efforts in our department are considered to be useful for hearing preservation.

It has been known that vestibular schwannoma could cause sudden or gradual hearing loss in its natural history [21,22,23]. Stangerup et al. reported that, while 53% of patients had good hearing and speech discrimination upon diagnosis, this proportion was 31% after ten years of observation [24]. Recently, Wasano et al. reported that in patients with vestibular schwannoma, there is a 25% probability that sensorineural hearing loss will recur within a year [25]. Conversely, there is also the possibility of worsening hearing loss owing to therapeutic interventions. Therefore, treatment strategy decisions must consider these two risks in every case. To decide when and how to intervene for patients with vestibular schwannomas, it is necessary to compare the risks of hearing loss in the natural course and hearing loss caused by surgery or radiation therapy. In this situation, the improved postoperative hearing preservation rate shown in this study may expand the indications for hearing preservation surgery as a treatment modality. Increasing the postoperative hearing preservation rate and performing surgery with higher precision will allow us to consider surgical intervention early when tumor growth is confirmed in patients with residual hearing. Namely, early surgery for vestibular schwannoma before hearing loss will become a possible choice.

Radiation therapy is thought to be beneficial for patients with vestibular schwannoma with relatively good hearing levels because radiation therapy is thought to be associated with a low risk of hearing loss. Short-term observation of patients after radiation therapy showed a good hearing preservation rate; however, recent long-term follow-up studies showed gradual hearing loss after radiation therapy, and serviceable hearing levels were lost in most cases within ten years [26,27,28,29]. There is another late-onset disadvantage of radiation therapy: the risk of malignant transformation of vestibular schwannoma [30,31]. Thus, it is difficult to apply radiation therapy to younger patients; however, it is still useful for relatively elderly patients. Compared with radiation therapy, surgical interventions have a disadvantage in that several patients have severe postoperative hearing loss by surgical interventions. These disadvantages have limited the application of surgery, especially for growing but smaller tumors. This study showed that hearing preservation after surgery was achieved in most of the selected cases. This might suggest that the disadvantages that limit the surgical indications may be overcome by our modification shown here. Moreover, although there is no difference in long-term QOL, short-term QOL within 5 years is slightly inferior to surgery or wait-and-scan compared to radiation therapy. The improved hearing preservation rate shown in this study may also contribute to improved short-term QOL after surgery [32]. Future studies with larger numbers of cases are warranted.

While we observed an improvement in the hearing preservation rate, several points should be addressed in future studies. In particular, we allowed partial resection in some cases, whereas total tumor resection has been preferred for long-term tumor control in previous studies. It has been reported that total tumor resection or nearly total resection showed a better local control rate of vestibular schwannoma than subtotal or partial resection. Subtotal resection is reportedly followed by tumor regrowth within two years in as many as 27.6% of patients [33,34], whereas the reported long-term recurrence rates of large vestibular schwannomas are less than 1% in 5 years after intended gross total resection through the retrosigmoid approach [35].

Our study revealed that a higher near-total resection rate was achieved under a surgical policy prioritizing hearing preservation in classes A and B. This result indicates that, if possible, surgery should be considered before ABR or OAE disturbance becomes obvious. In this study, we also revealed that in class C and D patients, tumor resection was limited to subtotal or partial resection in more patients. Whether this limitation of tumor resection could affect residual hearing levels after surgery, especially during long-term follow-up, should be carefully observed and examined in future studies.

## 5. Conclusions

In this study, we showed that an 80% hearing preservation rate could be achieved using our modified surgical approach with the retrolabyrinthine approach under modified reinforced continuous intraoperative monitoring for hearing preservation. This result suggests that early intervention by surgery could be considered before ABR and OAE disturbance are evident. In addition, our results may broaden the application of hearing preservation surgery for vestibular schwannoma.

## Figures and Tables

**Table 1:** Results of tumor resection extent. Total tumor resection (TR) or near total resection (NTR) was achieved in 18 of 33 patients (55%). In class A or B patients, TR or NTR was achieved in 10 of 15 patients (67%), whereas NTR was achieved in eight of 18 patients (44%) in class C or D. In contrast, relatively higher subtotal resection (STR) or partial resection (PR) rates were observed in class C or D cases.

		TR	NTR	STR	PR
Overall		2 (6%)	16 (48%)	12 (36%)	3 (9%)
Subgroup	A	2 (18%)	5 (45%)	3 (27%)	1 (9%)
	B		3 (75%)		1 (25%)
	C		2 (29%)	5 (71%)	
	D		6 (55%)	4 (36%)	1 (9%)
	A or B	2 (13%)	8 (53%)	3 (20%)	2 (13%)
	C or D	0 (0%)	8 (44%)	9 (50%)	1 (6%)

## Data Availability

Not applicable.
